# Correlation of Bariatric Surgery Effect on Lipid Profile Among Obese Patients

**DOI:** 10.7759/cureus.18118

**Published:** 2021-09-20

**Authors:** Mohammed Khaled S Zaki, Omamah H Al-Jefri, Reem E Kordi, Amal H Aljohani, Maha A Rizq, Ghaday H Kasem, Shahad B Abuasidah

**Affiliations:** 1 General Surgery, Taibah University, Madinah, SAU; 2 College of Medicine, Taibah University, Madinah, SAU; 3 Collage of Medicine, Taibah University, Madianh, SAU; 4 Collage of Medicine, Taibah University, Madinah, SAU

**Keywords:** total cholesterol, body mass index, high-density lipoprotein, overweight, obesity, sleevegastrectomy

## Abstract

Background

A considerable subpopulation of patients with morbid obesity present with dyslipidemia. It is characterized by elevated total cholesterol (TC), triglyceride (TG), low-density lipoprotein (LDL), and low high-density lipoprotein (HDL) concentrations. Sleeve gastrectomy (SG) is considered a method of treating morbid obesity and associated dyslipidemia.

Objective

To assess the effect of bariatric and metabolic surgery on lipid profile of morbidly obese patients.

Methods

We used a retrospective approach analyzing the lipid profiles of patients who underwent SG between January 2018 and July 2020. Patients were enrolled according to age (>17 years), pre-operative body mass index (BMI; >30 kg/m^2^), undergoing SG, and having complete follow-up records of lipid profiles. Baseline and post-operative lipid profiles, their variation, and the percentage of variation were compared.

Results

We analyzed data of 163 patients who underwent SG. The mean age was 36.75 ± 10.75 years, the mean BMI was 45.66 ±8.46, and the mean pre-operative TC, LDL, HDL, and TG were 4.67 ± 1.02, 2.55 ± 1.1, 1.14 ± 0.32, and 1.5 ± 1.11, respectively. There was a significant change in the mean level of TG as it was significantly higher pre-operatively compared to its mean level post-operatively. Furthermore, a significant change was observed in HDL. There was a non-significant change in levels of TC and LDL post-operatively.

Conclusion

SG showed to significantly reduce TG and elevate HDL in morbidly obese patients. On the contrary, TC and LDL were non-significantly affected. Further studies with longer follow-up are warranted to provide more reliable evidence.

## Introduction

Obesity and overweight are currently considered as a pandemic that is growing rapidly [1]. World Health Organization (WHO) recognized obesity in 2016 as a health burden, with 650 million of the worldwide population aged 18 years and above considered obese [2]. Obesity, in one way, is expressed as the relation between height and weight or BMI [3]. WHO defines overweight as a body mass index (BMI) ≥25 kg/m^2^ and obesity as a BMI ≥30 kg/m^2^ [3].

There are multiple factors that have a direct role in the pathophysiology of excess body weight including environmental, psychological, and hormonal factors, as well as genetics and lifestyle. The most common cause of obesity is the imbalance between calories consumed and their expenditure during physical activities [2]. Growth hormone (GH) and insulin-like growth factor (IGF-1) play a major role in regulating the metabolism of lipids. With the increase in BMI, the GH decreases consequently affecting lipid metabolism that leads to cardiovascular disease [2]. This sequence of dysfunctions leads to atherogenic dyslipidemia, which is characterized by high levels of triglycerides (TG) and low-density lipoprotein (LDL), and a low level of high-density lipoprotein (HDL) [1]. The National Cholesterol Education Program (NCEP) recommends healthy patients with no risk of coronary artery disease keep their serum levels of LDL cholesterol below 130 mg/dl, serum total cholesterol (TC) below 200 mg/dl, and serum TG below 150 mg/dl to avoid having a high risk of atherosclerotic cardiovascular diseases [4]. A previous study showed that for every 1 kg body weight loss, serum LDL cholesterol level drops by 0.02 mmol/L, serum TC level drops by 0.05 mmol/L, and serum HDL cholesterol level increases by 0.009 mmol/L [5]. In concordance, other studies showed that associated risk factors of cardiovascular disease, hypertension (HTN), type 2 diabetes mellitus (DM), and dyslipidemia, are directly related to higher severity of obesity, mortality, and morbidity [2,6,7]. Bariatric surgery is an effective treatment for morbid obesity, especially with comorbid diseases [2] as it leads to improvement in several cardiovascular risk factors such as DM, HTN, hypertriglyceridemia as well as raising the low HDL cholesterol [2,6]. While some researchers reported improvement in all parameters of the lipid profile after bariatric surgery, other researchers reported that the reduction was only in the levels of LDL cholesterol and serum TG, whereas there were non-significant changes in TC and HDL cholesterol levels [8,9]. Bariatric surgery is one of the most effective ways for achieving significant weight loss in a short time and is considered a treatment for obesity-associated co-morbidities, such as type 2 DM, HTN, and hyperlipidemia [10]. The most commonly used techniques of bariatric surgeries are sleeve gastrectomy (SG) and Roux-en-Y gastric bypass [11]. SG is an irreversible procedure where the surgeon removes half or more of the stomach and leaves a narrow sleeve. The Roux-en-Y gastric bypass procedure is both a restrictive as well as a malabsorptive procedure, where the stomach is reduced to a small pouch then connected directly to the small intestine [5]. In the Kingdom of Saudi Arabia (KSA), obesity and overweight affect more than 75% of the general population and it is estimated that approximately 15,000 bariatric operations are performed annually in the country [7,10]. Research showed that in KSA, SG is the most commonly performed bariatric surgery followed by Roux-en-Y gastric bypass [8]. Our study aims at the assessment of the effect of bariatric surgery on lipid profile in patients who underwent SG in Al-Madinah, KSA.

The aim of the study is to assess the effect of bariatric surgery on the lipid profile of both obese and morbidly obese patients.

## Materials and methods

Study design and time frame

This is a retrospective study analyzing the lipid profiles of patients who underwent SG between January 2018 and July 2020.

Study participants and settings

The participants were all patients who underwent SG during the study designated period, at the General Surgery department of King Fahad Hospital (KFH) in Madinah, KSA.

Data collection

The data of those patients were obtained from the Medical Administration Department under General Surgery Department.

Sampling methodology

A total sample size of 163 patients who underwent SG was collected in July 2020. The inclusion criteria were patients aged 18 years and above, patients who had a pre-operative BMI of ≥30 kg/m^2^, and those who had a follow-up record in KFH medical record system. Patients with incomplete follow-up records were excluded.

Measurement tools

A review of medical records was conducted to obtain data on lipid profile measurements (TC, HDL cholesterol, LDL cholesterol, and TG) before and after bariatric surgery.

Ethical considerations

An IRB ethical approval was obtained from the Institutional Review Board of King Fahad Hospital - Medina and Hospital Medical Administration Department at King Fahad Hospital. Information were only to be used by authors of the study and statistician. The confidentiality of patients’ data was preserved.

Statistical analysis

Data were analyzed using the Statistical Package for Social Sciences (SPSS) statistical analysis program version 25. Quantitative data were expressed as mean and standard deviation (mean ± SD), where Mann-Whitney and Wilcoxon tests were applied for non-parametric variables. Qualitative data were expressed as numbers and percentages. Spearman’s correlation analysis was used and a p-value of <0.05 was considered statistically significant.

## Results

Table 1 shows that 69.9% of patients of this study were females and 10.4% had comorbidities such as diabetes mellitus and cardiovascular disease. The mean age was 36.75 ± 10.75, the mean BMI was 45.66 ± 8.46 and the mean pre-operative TC, LDL, HDL, and TG were 4.67 ± 1.02, 2.55 ± 1.1, 1.14 ± 0.32, and 1.5 ± 1.11, respectively.

**Table 1:** Distribution of studied patients according to their age, gender, BMI, pre-operative levels of cholesterol, LDL, HDL, and TG. BMI: body mass index, LDL: low-density lipids, HDL: high-density lipids, TG: triglycerides.

Variable		Value	%
Gender	Male	49	30.1%
	Female	114	69.9%
Comorbidity	Present	17	10.4%
	Absent	146	89.6%
Variable		Mean	SD
Age		36.75	±10.75
BMI		45.66	±8.46
Pre-operative	Cholesterol	4.67	±1.02
	LDL	2.55	±1.1
	HDL	1.14	±0.32
	TG	1.5	±1.11
Post-operative	Cholesterol	4.61	±0.94
	LDL	3.09	±2.15
	HDL	1.22	±0.43
	TG	1.07	±0.58
Changes in serum level	Cholesterol	0.03	±0.8
	LDL	0.007	±0.92
	HDL	0.07	±0.31
	TG	0.42	±0.86

**Figure 1:**
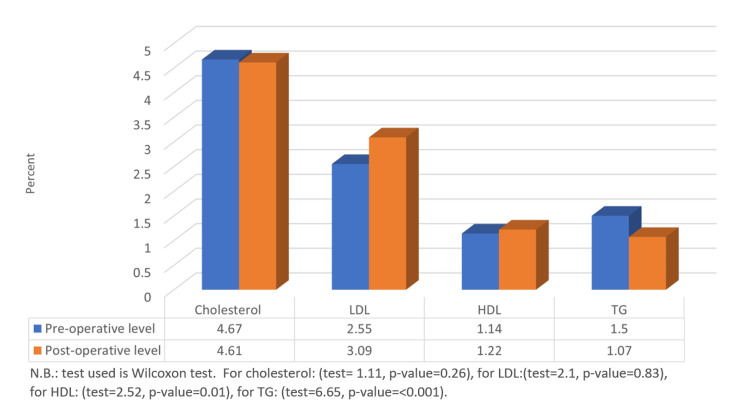
Comparison between pre and post-operative levels of cholesterol, LDL, HDL, and TG. LDL: low-density lipids, HDL: high-density lipids, TG: triglycerides.

Figure 1 shows a statistical comparison between pre-operative and post-operative lipid profile components. There was a significant change in the mean level of triglycerides and HDL comparing pre-operative and post-operative results (1.5 ± 1.11 vs 1.07 ± 0.58, 1.14 ± 0.32 vs 1.22 ± 0.43, respectively) which was statistically significant (p≤0.05). On the other hand, a non-significant change pre and post-operatively was observed for TC and LDL levels (p≥0.05).

Using Spearman’s correlation, changes in serum levels of TC, LDL, HDL, and TG were non-significantly correlated to patient’s age (Table 2), pre-operative BMI (Table 3), patients’ gender (Table 4), or comorbidities (Table 5).

**Table 2:** Spearman’s correlation between patients’ age and changes in serum level of cholesterol, LDL, HDL, and TG. LDL: low-density lipids, HDL: high-density lipids, TG: triglycerides.

Variable	Age	
	r	p-value
Changes in serum level of cholesterol	0.06	0.45
Changes in serum level of LDL	0.51	0.12
Changes in serum level of HDL	0.14	0.11
Changes in serum level of TG	0.13	0.1

**Table 3:** Spearman’s correlation between patients’ BMI and changes in serum level of cholesterol, LDL, HDL, and TG. LDL: low-density lipids, HDL: high-density lipids, TG: triglycerides.

Variable	BMI	
	r	p-value
Changes in serum level of cholesterol	0.04	0.58
Changes in serum level of LDL	0.11	0.76
Changes in serum level of HDL	0.1	0.28
Changes in serum level of TG	0.11	0.19

**Table 4:** Correlation between patients’ gender and changes in serum level of cholesterol, LDL, HDL, and TG. LDL: low-density lipids, HDL: high-density lipids, TG: triglycerides.

Variable	Gender		p-value
	Male	Female	
Changes in serum level of cholesterol	0.05 ± 0.71	0.03 ± 0.84	0.56
Changes in serum level of LDL	0.29 ± 0.9	0.7 ± 0.61	0.12
Changes in serum level of HDL	0.08 ± 0.26	0.06 ± 0.33	0.7
Changes in serum level of TG	0.58 ± 1.23	0.36 ± 0.64	0.77

**Table 5:** Correlation between patients’ comorbidity and changes in serum level of cholesterol, LDL, HDL, and TG.

Variable	Comorbidity		p-value
	Present	Absent	
Changes in serum level of cholesterol	0.15 ± 0.68	0.02 ± 0.81	0.45
Changes in serum level of LDL	0.07 ± 0.35	0.03 ± 1.21	0.86
Changes in serum level of HDL	0.23 ± 0.37	0.05 ± 0.3	0.16
Changes in serum level of TG	0.72 ± 1.03	0.39 ± 0.84	0.22

## Discussion

Obesity is considered nowadays a worldwide epidemic, and it is associated with different comorbidities, mostly related to dyslipidemia. Obesity-associated dyslipidemia most commonly includes elevated TG and LDL cholesterol, with decreased HDL cholesterol. The goal of this study is to review and compare lipid markers before and after bariatric surgery to detect changes in dyslipidemia parameters.

In the current study, there was a significant change in the mean level of TG as it was significantly higher pre-operatively compared to its mean level post-operatively (1.5 ± 1.11 vs 1.07 ± 0.58, respectively; p≤0.05). These findings are similar to Lira et al., which showed a reduction in serum TG levels after SG. A 40% reduction was observed at 24 months follow-up post-operatively [12]. Also, Hussein, found that TG levels were significantly lower after SG with no relation to age or sex of the enrolled patients, with a mean difference of 28.8 and a highly significant p-value (<0.001) [13]. This is consistent with the findings of this study, as there was a non-significant correlation between patients' age or gender and changes in serum level of TG (p≥0.05). A cohort study by Strain et al., found a reduction in TG one year after SG, with pre-operative mean 128.7 ± 66.7 mg/dl, which dropped after one year to 97.1 ± 43.5 mg/dl [14]. On the other hand, Szczuko et al. reported a temporary rise in the TG levels one-month post-operatively and a non-significant reduction after 12 months [15].

In the current study, there was no statistically significant change in levels of TC and LDL cholesterol. TC mean pre-operative level was 4.67 ± 1.02 compared to 4.61 ± 0.94 post-operatively (p≥0.26), while LDL cholesterol mean pre-operative level was 2.55 ± 1.1 compared to 3.09 ± 2.15 post-operatively (p≥0.83). Ruiz-Tovar et al. showed results similar to our study in which there was no statistical significance in the comparison between TC and LDL cholesterol in the pre-operative period and after 12 months of follow-up after SG [16]. Bužga et al. reported a non-significant change in serum levels of LDL [17].

Hussein reported a significant reduction at one year post-operative with a highly significant p-value (<0.001) and a mean difference of 26.2 mg/dl [13]. A meta-analysis by Heffron et al. mentioned that there was a highly significant reduction in mean TC at one year post-operatively compared to baseline (P<0.00001) [18]. A similar reduction in TC and LDL cholesterol was reported by Jamal et al., who evaluated 248 with a six-year follow-up, by 27% and 40%, respectively [19].

In the current study, mean levels of pre-operative HDL were significantly lower compared to their mean post-operatively (1.14 ± 0.32 vs 1.22 ± 0.43, respectively; p≤ 0.05). These findings are similar to those reported by Tovar et al., who reported a 28% increase in HDL [16]. Heffron et al., in their cohort study, showed similar results where a statistically significant increase in mean HDL cholesterol was detected one year post-operatively compared to baseline level (P<0.00001) [18]. Hady et al. and some other studies showed a non-significant increase in HDL cholesterol serum levels after SG [20].

Limitations of the current study include incomplete documentation of all components of lipid profile before or after the operation; there were two types of bariatric surgery done and our focus was only on SG, all the patients were consecutive and from the same center.

## Conclusions

The present study has shown that bariatric surgery is an effective modality for achieving significant weight loss and treatment for obesity and its co-morbidities. Our findings showed a significant change in the mean level of TG as it was significantly higher pre-operatively, and HDL were significantly lower compared to their mean post-operatively. On the other hand, a non-significant change was observed in TC and LDL levels.
